# Assessing the Distribution of Exotic Egg Parasitoids of *Halyomorpha halys* in Europe with a Large-Scale Monitoring Program

**DOI:** 10.3390/insects12040316

**Published:** 2021-04-01

**Authors:** Livia Zapponi, Francesco Tortorici, Gianfranco Anfora, Simone Bardella, Massimo Bariselli, Luca Benvenuto, Iris Bernardinelli, Alda Butturini, Stefano Caruso, Ruggero Colla, Elena Costi, Paolo Culatti, Emanuele Di Bella, Martina Falagiarda, Lucrezia Giovannini, Tim Haye, Lara Maistrello, Giorgio Malossini, Cristina Marazzi, Leonardo Marianelli, Alberto Mele, Lorenza Michelon, Silvia Teresa Moraglio, Alberto Pozzebon, Michele Preti, Martino Salvetti, Davide Scaccini, Silvia Schmidt, David Szalatnay, Pio Federico Roversi, Luciana Tavella, Maria Grazia Tommasini, Giacomo Vaccari, Pietro Zandigiacomo, Giuseppino Sabbatini-Peverieri

**Affiliations:** 1 Centro Ricerca e Innovazione, Fondazione Edmund Mach (FEM), via Mach 1, 38098 S. Michele all’Adige, TN, Italy; livia.zapponi@fmach.it (L.Z.); gianfranco.anfora@unitn.it (G.A.); 2 Dipartimento di Scienze Agrarie, Forestali e Alimentari, University di Torino (UniTO), Largo Paolo Braccini 2, 10095 Grugliasco, TO, Italy; francesco.trt@gmail.com (F.T.); silvia.moraglio@unito.it (S.T.M.); luciana.tavella@unito.it (L.T.); 3 Centro Agricoltura Alimenti Ambiente (C3A), Università di Trento, Via Mach 1, 38098 S. Michele all’Adige, TN, Italy; 4 Fondazione per la Ricerca l’Innovazione e lo Sviluppo Tecnologico dell’Agricoltura Piemontese (AGRION), Via Falicetto 24, 12100 Manta, CN, Italy; s.bardella@agrion.it; 5 Servizio Fitosanitario Emilia-Romagna, via Andrea da Formigine 3, 40128 Bologna, Italy; massimo.bariselli@regione.emilia-romagna.it (M.B.); alda.butturini@regione.emilia-romagna.it (A.B.); 6 Servizio Fitosanitario Friuli Venezia Giulia (ERSA), via Sabbatini 5, 33050 Pozzuolo del Friuli, UD, Italy; luca.benvenuto@ersa.fvg.it (L.B.); iris.bernardinelli@ersa.fvg.it (I.B.); giorgio.malossini@ersa.fvg.it (G.M.); 7 Consorzio Fitosanitario di Modena, via Santi 14, Direzionale Cialdini 1, 41123 Modena, Italy; stefano.caruso@regione.emilia-romagna.it (S.C.); giacvac@gmail.com (G.V.); 8 Consorzio Fitosanitario di Piacenza, via Cristoforo Colombo 35, 29122 Piacenza, Italy; ruggero.colla@regione.emilia-romagna.it; 9 Dipartimento di Scienze della Vita, Università di Modena e Reggio Emilia (UniMORE), Via G. Amendola 2, 42122 Reggio Emilia, Italy; elecosti@unimore.it (E.C.); emanuele.dibella@unimore.it (E.D.B.); lara.maistrello@unimore.it (L.M.); 10 Servizio Fitosanitario Lombardia (ERSAF), via Pola 12, 20124 Milano, Italy; paolo_culatti@regione.lombardia.it; 11 Centro di Sperimentazione Laimburg, Laimburg 6, 39051 Pfatten/Vadena, BZ, Italy; Martina.Falagiarda@laimburg.it (M.F.); Silvia.Schmidt@laimburg.it (S.S.); 12 CREA, Research Centre for Plant Protection and Certification, via di Lanciola 12a, 50125 Firenze, Italy; lucrezia.giovannini@crea.gov.it (L.G.); leonardo.marianelli@crea.gov.it (L.M.); piofederico.roversi@crea.gov.it (P.F.R.); 13 CABI, Rue des Grillons 1, 2800 Delemont, Switzerland; t.haye@cabi.org; 14 Dipartimento delle Finanze e dell’Economia, Servizio Fitosanitario Cantonale, Sezione dell’Agricoltura, Viale S. Franscini 17, 6500 Bellinzona, Switzerland; cristina.marazzi@ti.ch; 15 Department of Agronomy Food Natural Resources Animals and Environment, Università degli Studi di Padova (UniPD), Viale dell’Università 16, 35020 Legnaro, PD, Italy; alberto.mele@studenti.unipd.it (A.M.); alberto.pozzebon@unipd.it (A.P.); davide.scaccini@phd.unipd.it (D.S.); 16 Condifesa Lombardia Nord-Est, Servizio Tecnico, via Malta 12, 25124 Brescia, BS, Italy; lorenza.michelon@alice.it; 17 ASTRA, Astra Innovazione e Sviluppo srl, via Tebano 45, 48018 Faenza, RA, Italy; michele.preti@astrainnovazione.it; 18 Fondazione Fojanini, via Valeriana 32, 23100 Sondrio, SO, Italy; msalvetti@fondazionefojanini.it; 19 Strickhof, Fachstelle Obst, Riedhofstrasse 62, 8408 Winterthur, Switzerland; david.szalatnay@strickhof.ch; 20 CRPV, Centro Ricerche Produzioni Vegetali, via dell’Arrigoni 120, 47522 Cesena, FC, Italy; mgtommasini@crpv.it; 21 Department of Agricultural, Food, Environmental and Animal Sciences, Università di Udine (UniUD), Via delle Scienze 206, 33100 Udine, UD, Italy; pietro.zandigiacomo@uniud.it

**Keywords:** biological control, BMSB, exotic biological control agents, invasive species, natural enemies, *Trissolcus japonicus*, *Trissolcus mitsukurii*

## Abstract

**Simple Summary:**

The management of invasive alien species is a very challenging task. For the brown marmorated stink bug (*Halyomorpha halys*), classical biological control has been identified as the most suitable method to sustainably reduce its populations in the long-term. Among its natural enemies, two species were identified as the most promising candidates for biocontrol, *Trissolcus japonicus* and *Trissolcus mitsukurii*. Populations of these two species have recently been detected in Europe and to assess their distribution, a large-scale study was performed. Combining several monitoring methods, in four months (May–September 2019), a wide area covering northern Italy and parts of Switzerland was surveyed. The results showed that both species have spread into all types of habitats where *H. halys* is present and the parasitization of native species was rarely observed. Among native species, *Anastatus bifasciatus* was the predominant parasitoid of *H. halys*. This study supported the development of the first release program of *Tr. japonicus* in Europe.

**Abstract:**

The brown marmorated stink bug *Halyomorpha halys* is an invasive agricultural pest with a worldwide distribution. Classical biological control has been identified as the most promising method to reduce the populations of *H. halys*. Adventive populations of two candidates for releases, *Trissolcus japonicus* and *Trissolcus mitsukurii*, have recently been detected in Europe. To assess their distribution and abundance, a large-scale survey was performed. From May to September 2019, a wide area covering northern Italy and parts of Switzerland was surveyed, highlighting the expanding distribution of both *Tr. japonicus* and *Tr. mitsukurii.* Within four years after their first detection in Europe, both species have rapidly spread into all types of habitats where *H. halys* is present, showing a wide distribution and continuous expansion. Both exotic *Trissolcus* showed high levels of parasitism rate towards *H. halys*, while parasitization of non-target species was a rare event. The generalist *Anastatus bifasciatus* was the predominant native parasitoid of *H. halys*, while the emergence of native scelionids from *H. halys* eggs was rarely observed. The presence of the hyperparasitoid *Acroclisoides sinicus* was also recorded. This study provided fundamental data that supported the development of the first inoculative release program of *Tr. japonicus* in Europe.

## 1. Introduction

The brown marmorated stink bug *Halyomorpha halys* (Stål) (Hemiptera: Pentatomidae) is an invasive species from Central Asia that nowadays is present in North and South America [1,2], Europe [3] and the Caucasus [4], causing severe damage on many agricultural crops [5]. Due to the negative effects of frequent use of broad-spectrum insecticides (e.g., disruption of Integrated Pest Management, pesticide resistance), biological control has been identified as the most promising mid- and long-term solution [5]. Both the use of native natural enemies (inoculation and inundation biological control) and the more promising introduction of exotic *H. halys* egg parasitoids from Asia (classical biological control) are among the key management strategies for sustainable control of the invasive pest [6,7,8].

Support for biological control has regained momentum [9], however the debate on its environmental safety continues. Strict regulations are in place to limit the potential impact on biodiversity, and several international organizations have contributed to the development of step-by-step procedures for risk analysis, import and release of exotic agents [10,11]. However, the increased focus on unpredicted negative effects has contributed to the observed decline of classical biological control application and not without consequences: inaction or delay in the control of an invasive pest are in fact associated with increased pesticide use, impacting society and ecosystem dynamics [12]. Non-target effects, a critical aspect in classical biological control, can be predicted using host specificity testing, supporting decision making and reducing potential risks using cost/benefit analysis [13]. In North America and Europe, natural enemies that attack *Halyomorpha halys* include egg parasitoids that belong primarily to three Hymenopteran families: Scelionidae (*Telenomus* spp., *Trissolcus* spp., *Gryon* spp.), Eupelmidae (*Anastatus* spp.) and Encyrtidae (*Ooencyrtus* spp.), and generalist predators that feed on eggs and young nymphs [6]. In Europe, the egg parasitoid *Anastatus bifasciatus* (Geoffroy) (Hymenoptera: Eupelmidae) has been identified as the only native egg parasitoid capable of successfully developing in *H. halys*, thus it has been considered as a candidate for augmentative biological control releases [14,15]. However, experimental field trials involving sentinel egg masses showed a limited impact of the augmentative releases of *An. bifasciatus* [16], slightly higher in field studies with eggs laid in exclusion cages [17].

Recently, adventive populations of the main candidate for classical biological control, the Asian egg parasitoid *Trissolcus japonicus* (Ashmead) (Hymenoptera: Scelionidae), were detected both in North America [18] and in Europe, including Italy [19,20] and Switzerland [21]. In addition, adventive populations of another Asian egg parasitoid, *Trissolcus mitsukurii* (Ashmead), were detected on *H. halys* egg masses in Italy [19,20,22,23]. The scattered distribution of the two exotic *Trissolcus* species in Italy and Switzerland might have been caused by multiple introductions on the same pathways of entry of *H. halys*, as parasitized egg masses on plants or diapausing adults [21]. Furthermore, their post-introduction dispersal ability may have further contributed, as was proposed for *Tr. japonicus* in North America [24]. The presence of the two exotic egg parasitoids in Europe suggests that there is the hope for a future biological control solution for the management of *H. halys*. However, to date, the current distribution of both exotic *Trissolcus* species and their impact on *H. halys* populations in Italy and Switzerland are poorly understood. Considering the high expectations on their potential for controlling the pest, field investigations on the status of the adventive populations have been considered mandatory. In addition, knowledge of the current incidence and distribution will provide a baseline for future studies, investigating movement, spread, and impact of these parasitoids on *H. halys* and non-target species.

The objective of this study was to evaluate the distribution of the adventive populations of *Tr. japonicus* and *Tr. mitsukurii* in North Italy and Switzerland in a large-scale monitoring program. In addition, their parasitism rate, impact on non-target species, phenology and habitat were evaluated. Lastly, the collected data were used to support the petition for the final approval of *H. halys* biological control program and the release in the field of laboratory reared *Tr. japonicus.*

## 2. Materials and Methods

### 2.1. Field Surveys

With the aim to cover a wide geographical area, a network of 21 partners from northern Italy and Switzerland was developed, including research institutes, universities and local phytosanitary services. The members of the consortium jointly developed the workplan and survey protocol, in order to standardize all the key steps (e.g., data recording, sample storing, and taxonomic identification).

Field surveys were performed from late May to mid-September 2019 and took place in agricultural, urban and semi-natural habitats. To increase the sampling coverage, several types of surveys were combined: (a) visual inspection in fixed locations, repeated at least three times during the season; (b) visual inspections in non-fixed locations where *H. halys* adults and nymphs were observed; (c) deployment of sentinel egg masses (see Table S1 for further details). For the first two survey methods, the underside of leaves of trees, shrubs and herbaceous plants were inspected, searching for egg masses of *H. halys* and other pentatomids, for at least 30 min. For sentinel egg masses, both fresh (24 h) and frozen (stored at −80 °C for maximum a month) egg masses were used and deployed for 3 to 5 days; for further details, see Stahl et al. [21] and Zapponi et al. [23].

After collection, each egg mass was labeled, stored separately, and transported back to the laboratories for successive rearing of nymphs and/or parasitoids. For each sample, date, surveyor code, GPS coordinates and habitat (type of crop and host plant) were recorded.

### 2.2. Laboratory Handling of Collected Egg Masses

Egg masses were stored in 90 mm plastic Petri dishes or 50 mL plastic vials and kept at a temperature of 24–26 °C and L16:D8 photoperiod. Samples were checked daily, until nymph or parasitoid emergence was completed. For the identification of native pentatomid egg masses, field-collected adults were reared by several co-authors, in order to have fresh laid egg masses that were then compared with the field-collected ones, to ensure their correct identification [25]. When a species could not be assigned, genus level was used.

Parasitoids were promptly transferred into ethanol and preserved for subsequent identification. For each egg mass, the total number of eggs, hatched nymphs and emerged parasitoids were recorded. All emerged parasitoids were counted and sexed.

To compare the parasitism rate of the most common parasitoid species, the number of parasitized eggs in an egg mass over the total number of intact eggs was calculated, excluding eggs that did not hatch and those that showed signs of predation [26]. The parasitism rate was calculated only on field-collected egg masses (data from sentinel egg masses were not included in this analysis). Furthermore, only egg masses from which a single parasitoid species had emerged were considered, to avoid underestimation/overestimation of parasitism rates.

### 2.3. Taxonomic Identification

Parasitoids were first separated and identified at family and genus levels. Then, for *Anastatus* the keys proposed by Askew and Nieves-Aldrey [27] were used, while for *Acroclisoides* the method of Sabbatini Peverieri et al. [28] was followed. Among Scelionidae, *Trissolcus* species were identified using Talamas et al. [29] and Tortorici et al. [30]. *Telenomus* species were identified, whenever possible, using Kozlov and Kononova [31] and comparing the specimens with the images of the primary types (kindly provided by Elijah Talamas).

### 2.4. Data Analysis

The occurrence of stink bug and exotic parasitoids was analyzed using QGIS 3.14 (Development Team 2018, QGIS Geographic Information System), mapping the presence of *Tr. japonicus* and *Tr. mitsukurii* on a heatmap obtained from *H. halys* occurrence data. Descriptive analyses were performed with R Statistical Software (v.3.5.2) [32].

## 3. Results

### 3.1. Field Survey

In four months, a total of 4348 and 285 egg masses of *H. halys* and of other pentatomids, respectively, were collected in an area of approximately 140,000 km^2^, covering northern Italy and parts of Switzerland (survey methods a and b). Most egg masses were found in cultivated areas (51.58%) or urban environments (44.81%), while fewer egg masses (5.21%) were collected in semi-natural habitats (Figure S1, content). About 93.85% of the collected egg masses belonged to *H. halys*, while the rest belonged to several other Pentatomidae species, including *Palomena prasina* (L.), *Rhaphigaster nebulosa* (Poda), *Piezodorus lituratus* (F.), *Dolycoris baccarum* (L.) and *Nezara viridula* (L.) (see Table 1).

### 3.2. Parasitoid Species Composition

From the collected pentatomid egg masses, the eupelmid *An. bifasciatus* emerged as well as numerous Scelionidae belonging to the genera *Trissolcus* and *Telenomus*, specifically: *Tr. basalis* (Wollaston), *Tr. belenus* (Walker), *Tr. cultratus* (Mayr), *Tr. japonicus*, *Tr. mitsukurii*, *Tr. semistriatus* (Nees von Esenbeck), *Tr. viktorovi* Kozlov, *Te. turesis* Walker, and *Telenomus* sp. (see Table 1 for hosts and number of parasitized egg masses). The hyperparasitoid *Acroclisoides sinicus* (Huang and Liao) (Hymenoptera: Pteromalidae) was also recorded, emerging from parasitized egg masses of *H. halys* and *N. viridula* (Table 1).

Considering only parasitized *H. halys* egg masses (974, representing 22.40% of total number of collected egg masses), the most common species that emerged were *An. bifasciatus*, *Tr. mitsukurii* and *Tr. japonicus*. Unhatched and predated eggs represented the 25.26% of the total number of eggs (21.51% for non-parasitized egg masses, 27.20% for parasitized egg masses). See paragraph 3.3 for results on the number of parasitized eggs per egg mass.

Concerning native pentatomid egg masses (88 parasitized egg masses in total), *Tr. japonicus* and *Tr. mitsukurii* emerged in low numbers. The only native species parasitized by *Tr. japonicus* was *Pa. prasina* (five egg masses, representing 13% of the parasitized *Pa. prasina* egg masses collected)*,* while *Tr. mitsukurii* emerged from egg masses of *D. baccarum* (2; 28% of parasitized *D. baccarum* egg masses), *N. viridula* (3; 10% of parasitized *N. viridula* egg masses), *Pa. prasina* (2; 5% of parasitized *Pa. prasina* egg masses), and from an egg mass ascribed to a pentatomid of the subfamily Asopinae.

### 3.3. Parasitism Rate and Phenology

**Figure 1:**
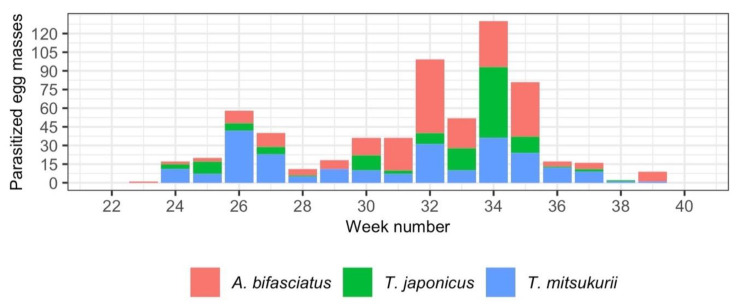
Number of parasitized egg masses collected throughout the sampling period (May–September 2019) for *Anastatus bifasciatus*, *Trissolcus japonicus* and *Trissolcus mitsukurii*.

Comparing the number of collected *H. halys* egg masses parasitized by the three most common species (Figure 1), parasitism by *Tr. mitsukurii* was higher in June, with two slightly lower peaks in August. Parasitism by *Tr. japonicus* showed a similar pattern, but it reached its maximum in August. These patterns were consistent across the study area.

**Figure 2:**
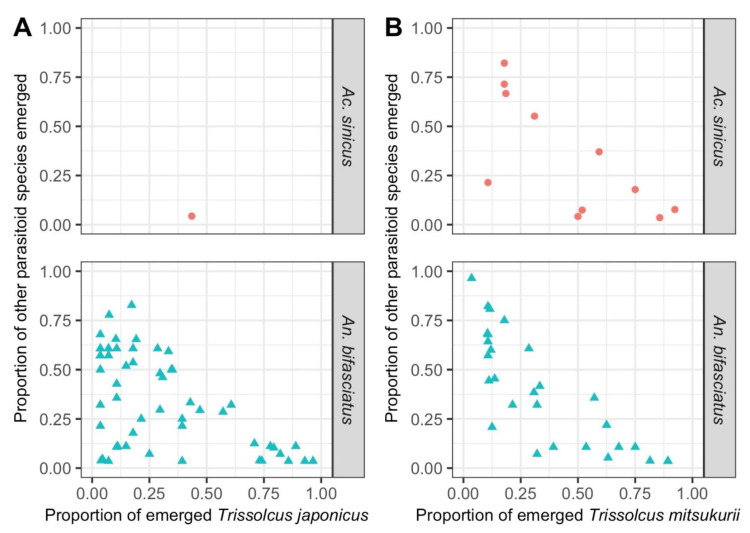
Multiparasitism of egg masses of *Halyomorpha halys* where the emergence of two species was observed (survey methods a and b): (**A**) proportion of emerged individuals for *Trissolcus japonicus* and other species (*Acroclisoides sinicus* and *Anastatus bifasciatus*) (N. of considered egg masses = 56); (**B**) proportion of emerged individuals for *Trissolcus mitsukurii* and other species (*Ac. sinicus* and *An. bifasciatus*) (N. of considered egg masses = 39).

Across the surveyed regions, the percentage of parasitized egg masses was very variable for the three most common parasitoids: 0.45–53.85% for *Tr. japonicus*, 0.17–20.20% for *Tr. mitsukurii* and 0.85–19.55% for *An. bifasciatus* (Table 2). The parasitism rate varied as well for *Tr. japonicus* (71.43–100%), *Tr. mitsukurii* (88.59–97.40%) and *An. bifasciatus* (3.57–76.09%). The emergence of more than one species from the same *H. halys* egg mass was observed multiple times (Figure 2), from 96 egg masses in total (*n* = 2539 total number of eggs), and in the 83.33% it involved combinations of *An. bifasciatus* and 1–2 species. *Anastatus bifasciatus* emerged often together with *Tr. japonicus* (*n* = 55) and *Tr. mitsukurii* (*n* = 28), while *Ac. sinicus* was less frequent (*n* = 12 on *Tr. mitsukurii* and *n* = 1 on *Tr. japonicus*). Two other associations were observed once: *Tr. cultratus* and *Tr. japonicus*; *Tr. basalis* and *Tr. mitsukurii*. The emergence of two species generally coincided with the lack of emergence if *H. halys* nymphs (only 1.5% of eggs) and the presence of unhatched eggs (25% of eggs).

### 3.4. Habitat, Host Species and Distribution

Both exotic parasitoids were found in agricultural and urban areas. In agricultural systems, parasitized egg masses were found in conventional, integrated pest management (IPM) and organic orchards and fields, both on the edge (e.g., perimetral shrubs, hedgerows) and in the inner part of the cultivated area. In urban areas, parasitized egg masses were located in gardens, parks, parking lots and street trees. Concerning host plants, *Tr. japonicus* was recorded on both cultivated plants (i.e., *Corylus avellana* L., *Prunus persica* (L.) Batsch, *Olea europaea* L.) and trees planted in hedgerows or along streets (i.e., *Acer* spp., *Acer campestre* L., *Catalpa bignonioides* Walter., *Fraxinus* spp., *Tilia platyphyllos* Scop.). See Table S2 for the complete list of *H. halys* plant host species.

*Trissolcus mitsukurii* was also recorded on cropland (on *Acer* spp., *Actinidia deliciosa* (A. Chev.) C.F. Liang and A.R. Ferguson, *Ailanthus altissima* (Miller) Swingle, *Corylus avellana* L., *Glycine max* (L.) Merr., *Malus domestica* (Suckow) Borkh., *Vitis vinifera* L., *Ziziphus jujuba* Mill.) and in urban areas (on *Acer* spp., *A. altissima*, *Fraxinus* spp., *Prunus spinosa* L., *Ziziphus jujuba* Mill.).

**Figure 3:**
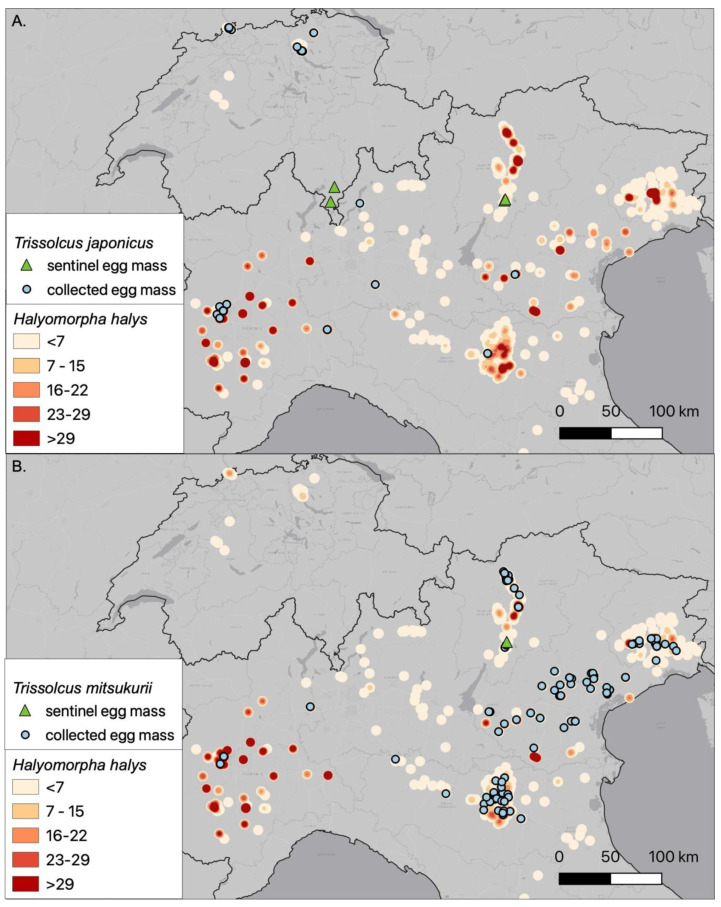
Distribution map of the collected egg masses for *Halyomorpha halys*, showing the emergence of the two exotic parasitoids in northern Italy and Switzerland (survey methods a, b and c): (**A**) *Trissolcus japonicus* and (**B**) *Trissolcus mitsukurii*. The heatmap is based on the abundance of collected egg masses of *H. halys* where orange reflect a low number and red a high number of egg masses. Sentinel egg mass records are derived from Stahl et al. [21] and Zapponi et al. [23].

The surveys (methods a, b and c) proved that both species are widely distributed in northern Italy while in Switzerland only *Tr. japonicus* is currently present (Figure 3). *Anastatus bifasciatus* and *Tr. mitsukurii* were recorded in all surveyed regions. The survey highlighted also that the distribution ranges of both exotic egg parasitoids are expanding southwards from the first Italian records (e.g., recording the first observation in Emilia-Romagna region).

## 4. Discussion

Overall, the results of the large-scale monitoring demonstrated a wide distribution and continuous expansion of *Tr. japonicus* and *Tr. mitsukurii*, compared to previous studies [19,20,22,23]. Four years following their first detection in Europe, both species have rapidly spread into all types of habitats where *H. halys* has been surveyed. Host plants included a wide range of crop and non-crop species. The patterns of occurrence suggest that *Tr. mitsukurii* is currently more widespread than *Tr. japonicus* and more abundant in the eastern part of northern Italy, while in Switzerland *Tr. mitsukurii* appears to be still absent. This different expansion could be explained by the timing of arrival of the two species and first locality of accidental introduction, since the earliest official record of *Tr. mitsukurii* for northern Italy was traced back to 2016 [22], two years before the earliest detection of *Tr. japonicus* [19]. Furthermore, the different abundance may be responsible also for the dissimilar observed habitat prevalence (agricultural/urban) observed for *Tr. japonicus* and *Tr. mitsukurii*. Whether other factors (e.g., host plant availability, sensitivity to chemicals) are responsible for the observed pattern merits further investigation.

As previously observed [13,14,20], our study confirmed that among native species, the generalist *An. bifasciatus* was the predominant parasitoid of *H. halys* in Italy, with a wide distribution and abundance. Conversely, the emergence of native scelionids was rarely observed. In the present study, *An. bifasciatus* emerged often from egg masses parasitized for other species as well. As previously pointed out [33,34] the role of *An. bifasciatus* (as competitor or hyperparasitoid) should be further investigated.

Yet, the lack of detected emergence is no evidence that an attempt of parasitization has not taken place. Molecular methods proved that native scelionids may frequently parasitize *H. halys* eggs but fail to develop, resulting in an evolutionary trap [35]. Similarly, laboratory trials showed that native *Trissolcus* species are able to induce egg abortion (non-reproductive effects) in *H. halys* [25]. Studies carried out on North American native parasitoids suggest that there is currently no evidence of intraspecific variation in traits that allow native parasitoids to successfully develop in *H. halys* eggs [36]. However, the successful adaptation of native parasitoids to a new host may occur over larger timescales (i.e., decades) [37] and should be further studied in Europe.

Field data from the native range of both *Trissolcus* species indicate that their host ranges are restricted to a few pentatomid species [7,38]. Both parasitoid species showed a favorable degree of specificity towards *H. halys* in the present study, even though they developed on other hosts (for example, *D. baccarum*, *N. viridula* and *Pa. prasina* for *Tr. mitsukurii*; *Pa. prasina* for *Tr. japonicus*), it was a relatively occasional event in this survey. Nevertheless, the data on native stink bugs were limited since the focus of the present study was on *H. halys*, and host plants of native pentatomids were only marginally sampled, resulting in a smaller number of collected egg masses. The use of sentinel egg masses produced in rearing facilities could overcome the potential for a relatively low number of collected samples, but unfortunately such egg masses are less reliable since they tend to underestimate parasitoid impact [39].

The actual impact on native stink bugs should be evaluated with specifically designed field experiments, which should take into account hidden trophic interactions [40] as well. Further investigations are needed to assess whether behavioral barriers (such as phenology, habitat preference and interspecific competition) exist and could prevent non-target parasitism in the field [41].

Both *Tr. japonicus* and *Tr. mitsukurii* showed notable levels of parasitism rate, which is helpful to build up populations. However, this first large-scale monitoring also showed a widespread presence of the hyperparasitoid *Ac. sinicus*, the expansion of which could cause possible problems in the future, limiting the impact of *H. halys* natural enemies. *Acroclisoides sinicus* was recently detected in many countries worldwide, but it is suspected to be an Asian species and its influence on the host–parasitoid trophic chain should be further investigated [28,42]. The generalist parasitoid *An. bifasciatus* was frequently found throughout the survey region, but its impact on *H. halys* is considered low [15] and inundative releases of this species did not result in sufficient pest control [16]. Accordingly, future efforts should focus on the two coevolved biological control agents originating from the pest’s area of origin.

Studies assessing the long-term trend of host–parasitoid interactions between Pentatomidae and Scelionidae are scarce [19,43] and several important issues remain unexplored (such as the variation of parasitism rate with time, for adventive populations). Further investigations on population and community ecology are needed to provide a strong basis for biological control prediction and management [44]. The present large-scale study (with shared survey protocols and analysis approaches) provides a baseline on parasitoid species composition and parasitism rate that may be used in the future to assess the impact of both adventive and released *Trissolcus* populations, and thus evaluate the output of classical biological control programs. In particular, data collected with the presented approach could also be used to understand whether laboratory predictions on non-target exploitation are consistent with what is observed in the field, in the area of introduction.

In Europe, the application of classical biological control is regulated by stringent risk assessments. Bureaucratic barriers, general criticisms due to poor public awareness, together with limited funding, may block the release of biological control agents [45,46]. As recently stated [47,48], more coordinated efforts are needed, with taxonomists and researchers working together, to create the grounds for effective biological area-wide control programs. The cooperative character of the present study was fundamental to cope with the difficult task of performing a large-scale survey in a limited amount of time, and provide data for *Tr. japonicus* risk assessment, essential for the ongoing release of the tested strain in Italy. The present approach could be applied to post-release studies as well, coping with logistically and economically challenging tasks. Biocontrol long-term development requires coordinated efforts, involving local and central governments, the engagement of the scientific community and public support [49]. Thus, creating the means for large scale international collaborations represents a further step towards the implementation of sustainable agriculture.

## 5. Conclusions

Small founder populations of *Tr. japonicus* and *Tr. mitsukurii* initially recorded in Switzerland [21] and in northern Italy [19,20,22,23] are currently expanding their range, following the path of their host *H. halys*. Bioclimatic models suggest that at least *Tr. japonicus* will be able to thrive and expand in all areas where *H. halys* already occurs, but also in nearly all areas where *H. halys* has been predicted to expand [50]. Considering that *H. halys* control still largely relies on the use of broad-spectrum insecticides, the reduced risk of potential non-target effects on a limited number of native stink bug species should not hamper future releases of *Tr. japonicus* [41], but should however be carefully evaluated with the support of a cost/benefit analysis [13]. The negative perceptions associated with classical biological control should not result in lost opportunities of increasing the sustainability of invasive pest management [12].

## Figures and Tables

**Table 1:** Number of egg masses collected (N. EM) for each stink bug species and number of parasitized egg masses (N. PEM), in total and in brackets for each emerged parasitoid and hyperparasitoid species (survey methods a and b).

Species	N. EM	N. PEM	Species of Parasitoids	Species of Hyperparasitoids
*Carpocoris* spp.	8	4	*Telenomus* sp. (4)	
*Dolycoris baccarum*	22	7	*Anastatus bifasciatus* (1), *Telenomus* sp. (3), *Trissolcus belenus* (1), *Trissolcus mitsukurii* (2)	
*Eurydema* spp.	4	2	*Trissolcus viktorovi* (2)	
*Halyomorpha halys*	4348	974	*Anastatus bifasciatus* (624)*, Telenomus* sp. (25)*, Trissolcus basalis* (20), *Trissolcus belenus* (4)*, Trissolcus cultratus* (14)*, Trissolcus japonicus* (152)*, Trissolcus mitsukurii* (325), *Trissolcus semistriatus* (4), Undet. (20)	*Acroclisoides sinicus* (69)
*Nezara viridula*	153	31	*Anastatus bifasciatus* (17)*, Trissolcus basalis* (11)*, Trissolcus mitsukurii* (3)	*Acroclisoides sinicus* (1)
*Palomena prasina*	74	38	*Anastatus bifasciatus* (11)*, Telenomus* sp. (7)*, Telenomus turesis* (2)*, Trissolcus cultratus* (11)*, Trissolcus japonicus* (5)*, Trissolcus mitsukurii* (2)	
*Pentatoma rufipes*	6	2	*Trissolcus cultratus* (2)	
*Piezodorus lituratus*	7	1	*Trissolcus semistriatus* (1)	
*Rhaphigaster nebulosa*	11	2	*Anastatus bifasciatus* (1)*, Telenomus* sp. (1)	

**Table 2:** Number of egg masses (N. EM) and eggs (N. E) for *Halyomorpha halys* collected in the different survey areas (methods a and b), showing percentage of parasitized egg masses (PEM) and parasitized eggs (PE), and resulting parasitism rate (PR) for *Trissolcus japonicus* (A), *Trissolcus mitsukurii* (B) and *Anastatus bifasciatus* (C), calculated on egg masses from which only one parasitoid species had emerged.

A. *Trissolcus japonicus*						
**Country**	**Region/Canton**	**N. EM**	**N. E**	**PEM (%)**	**PE (%)**	**PR (Mean ± SE)**
Italy	Piedmont	1788	46,824	4.03	2.98	89.43 ± 2.97
	Veneto	669	16,966	0.45	0.29	100.00 ± 0.00
Switzerland	Basel	26	625	53.85	50.88	80.25 ± 6.95
	Zürich	25	673	32.00	25.41	71.43 ± 10.19
B. *Trissolcus mitsukurii*						
**Country**	**Region/Canton**	**N. EM**	**N. E**	**PEM (%)**	**PE (%)**	**PR (Mean ± SE)**
Italy	Emilia Romagna	629	16,707	3.66	2.75	97.40 ± 1.68
	Friuli-Venezia Giulia	589	13,874	20.20	14.49	96.21 ± 1.38
	Piedmont	1788	46,824	0.17	0.09	91.23 ± 8.77
	Trentino-Alto Adige	585	15,506	5.98	4.15	96.70 ± 2.92
	Veneto	669	16,966	6.88	4.66	88.59 ± 3.27
C. *Anastatus bifasciatus*						
**Country**	**Region/Canton**	**N. EM**	**N. E**	**PEM (%)**	**PE (%)**	**PR (Mean ± SE)**
Italy	Emilia Romagna	629	16,707	19.55	2.36	66.06 ± 2.78
	Friuli-Venezia Giulia	589	13,874	0.85	0.35	44.89 ± 19.54
	Piedmont	1788	46,824	15.72	7.79	73.97 ± 1.92
	Trentino-Alto Adige	585	15,506	11.97	5.62	76.09 ± 3.69
	Veneto	669	16,966	1.05	0.62	68.32 ± 15.15
Switzerland	Basel	26	625	3.85	3.36	75.00
	Zürich	25	673	4.00	0.15	3.57

## Data Availability

The data that support the findings of this study are available from the corresponding author, upon request and agreement of the authors.

## Supplementary Materials

The following are available online at https://www.mdpi.com/article/10.3390/insects12040316/s1, Figure S1: Distribution of the collected *Halyomorpha halys* egg masses in Italy (IT) and Switzerland (CH), according to the different surveyed contexts: cropland, semi-natural habitats and urban areas, Table S1: Survey methods applied in the different areas: (a) visual inspection in fixed locations (FV), repeated at least three times during the sampling season; (b) visual inspection in non-fixed locations (V) and (c) deployment of sentinel egg masses (S). Table S2: Host plant for the collected *Halyomorpha halys* egg masses.
