# Supplementary material for: Assessing the Distribution of Exotic Egg Parasitoids of Halyomorpha halys in Europe with a Large-Scale Monitoring Program
Source: Insects. 2021 Apr 1;12(4):316. doi: 10.3390/insects12040316 (PMC8067160; doi:10.3390/insects12040316)
Supplement: Supplementary file 1 [file insects-12-00316-s001.pdf]

Supplementary Material: Assessing the Distribution of Exotic Egg Parasitoids of *Halyomorpha halys* in Europe with a Large-Scale Monitoring Program

Livia Zapponi, Francesco Tortorici, Gianfranco Anfora, Simone Bardella, Massimo Bariselli, Luca Benvenuto, Iris Bernardinelli, Alda Butturini, Stefano Caruso, Ruggero Colla, Elena Costi, Paolo Culatti, Emanuele Di Bella, Martina Falagiarda, Lucrezia Giovannini, Tim Haye, Lara Maistrello, Giorgio Malossini, Cristina Marazzi, Leonardo Marianelli, Alberto Mele, Lorenza Michelon, Silvia Teresa Moraglio, Alberto Pozzebon, Michele Preti, Martino Salvetti, Davide Scaccini, Silvia Schmidt, David Szalatnay, Pio Federico Roversi, Luciana Tavella, Maria Grazia Tommasini, Giacomo Vaccari, Pietro Zandigiacomo and Giuseppino Sabbatini-Peverieri

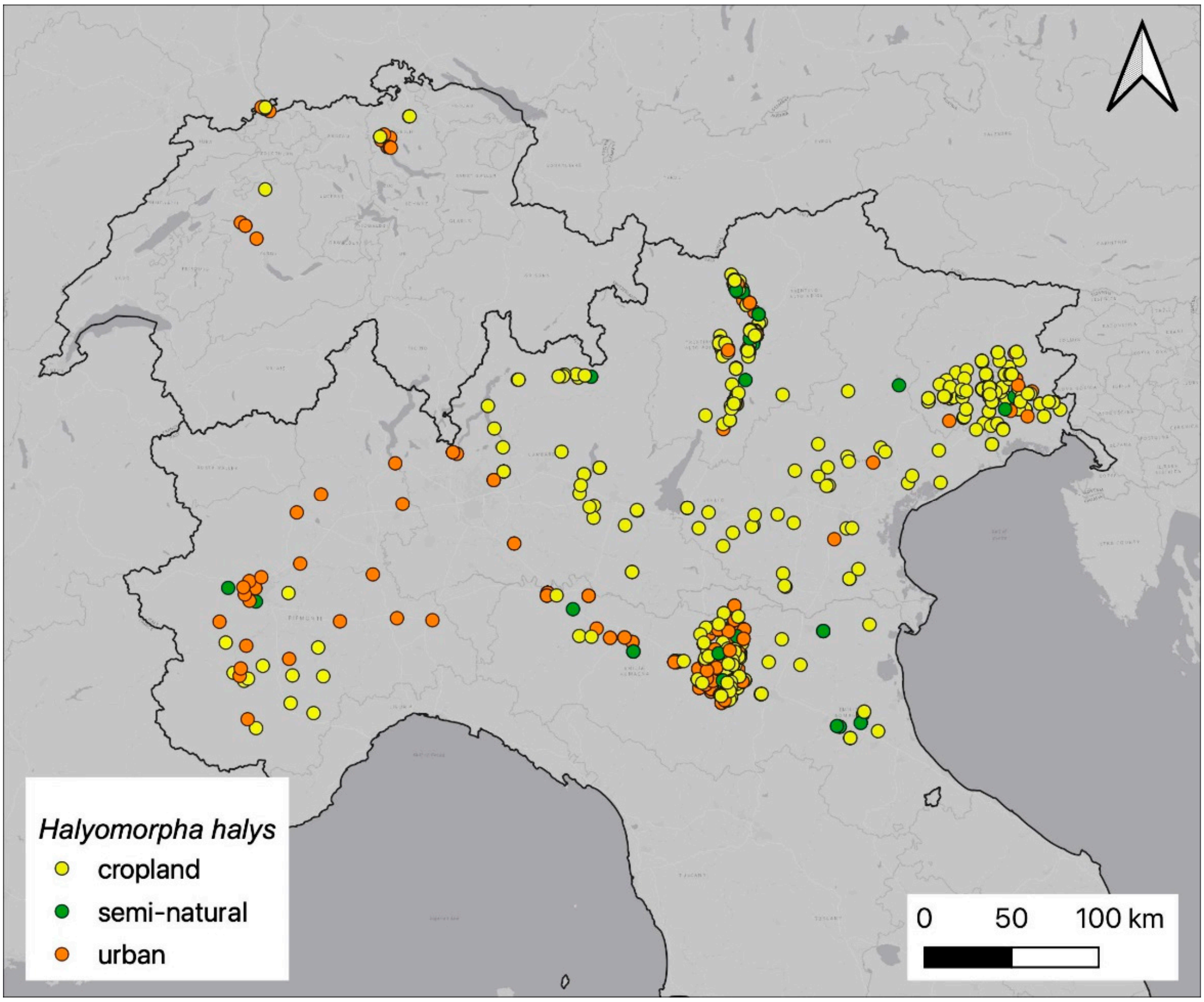

Figure S1. Distribution of the collected *Halyomorpha halys* egg masses in Italy (IT) and Switzerland (CH), according to the different surveyed contexts: cropland, semi-natural habitats and urban areas.

Table S1. Survey methods applied in the different areas: (a) visual inspection in fixed locations (FV), repeated at least three times during the sampling season; (b) visual inspection in non-fixed locations (V) and c) deployment of sentinel egg masses (S).

**Country**	**Region/Canton**	**Survey**
Italy	Emilia Romagna	FV, V
	Friuli-Venezia Giulia	V
	Lombardy	V
	Piedmont	FV
	Trentino-Alto Adige[1]	FV, V, S [1]
	Veneto	FV, V, S
Switzerland	Basel	V
	Bern	V
	Ticino	S [2]
	Zürich	V

[1] Zapponi et al., 2020; [2] Stahl et al., 2019.

Table S2. Host plant for the collected *Halyomorpha halys* egg masses.

**Host Plant Species**
Acer campestre
Acer negundo
Acer sp.
Actinidia sp.
Ailanthus altissima
Ocimum basilicum
Cannabis sp.
Carpinus betulus
Cornus mas
Cornus sanguinea
Corylus avellana
Crataegus monogyna
Diospyros kaki
Phaseolus vulgaris
Ficus carica
Fraxinus sp.
Geranium sp.
Helianthus annuus
Hedera helix
Juglans sp.
Laburnum anagyroides
Lactuga sp.
Liquidambar sp.
Malus domestica
Paulownia tomentosa
Solanum lycopersici
Prunus armeniaca
Prunus avium
Prunus cerasus
Prunus domestica
Prunus padus
Prunus persica
Prunus sp.
Prunus spinosa
Punica granatum
Sambucus nigra
Sorbus aria
Syringa sp.

Tilia platyphyllos
Tilia sp.
Viburnum lantana
Vitis vinifera
Ziziphus jujuba

References

1. Stahl, J.; Tortorici, F.; Pontini, M.; Bon, M.C.; Hoelmer, K.; Marazzi, C.; Tavella, L.; Haye, T. First discovery of adventive populations of *Trissolcus japonicus* in Europe. *J. Pest Sci.* **2019**, *92*, 371–379. https://doi.org/10.1007/s10340-018-1061-2
2. Zapponi, L.; Bon, M.C.; Fouani, J.M.; Anfora, G.; Schmidt, S.; Falagiarda, M. Assemblage of the egg parasitoids of the invasive stink bug *Halyomorpha halys*: insights on plant host associations. *Insects*, **2020,** *11*, 588. https://doi.org/10.3390/insects11090588
